# A 20-Year Single Center Experience of Right Lateral Sector Graft in Adult Living Donor Liver Transplantation With Special Reference to Biliary Complication

**DOI:** 10.3389/ti.2025.14606

**Published:** 2025-07-02

**Authors:** Tomoaki Hayakawa, Nobuhisa Akamatsu, Takashi Kokudo, Kyoji Ito, Yujiro Nishioka, Yujiro Mihara, Akihiko Ichida, Takeshi Takamoto, Yoshikuni Kawaguchi, Kiyoshi Hasegawa

**Affiliations:** Artificial Organ and Transplantation Surgery Division, Department of Surgery, Graduate School of Medicine, University of Tokyo, Tokyo, Japan

**Keywords:** biliary stricture, living donor liver transplantation (LDLT), right lateral sector graft, donor pool expansion, vascular complication

## Abstract

Right lateral sector grafts (RLSGs) in living donor liver transplantation (LDLT) expand donor options, however, their long-term outcomes and complication rates remain unclear. We analyzed 661 LDLTs (42 RLSGs, 363 right liver grafts, 243 left liver grafts, and 13 left lateral section grafts) performed between 2000 and 2021 at the University of Tokyo Hospital. RLSG donors experienced a 4.8% major complication (Clavien-Dindo grade ≥3b) rate with no mortality. RLSG recipients had a 38.1% major complication rate and a 9.5% 90-day mortality rate. Compared with other graft types, RLSG recipients had higher rates of hepatic artery thrombosis (9.5% vs. 3.1%), portal vein stenosis (14.3% vs. 1.9%), and biliary stricture (42.9% vs. 16.3%). The 5-year survival rate for RLSG recipients (79.2%) did not differ significantly from other graft types (84.7%). Graft bile ducts measuring >4 mm were associated with increased anastomotic biliary stricture. RLSG, the only option for 33 recipients, expanded the donor pool by 5%. Although RLSG is associated with higher vascular and biliary complication rates, it demonstrates favorable long-term survival and significantly expands the donor pool. For patients without suitable conventional graft options, RLSG represents a viable choice that provides life-saving transplantation opportunities.

## Graphical Abstract

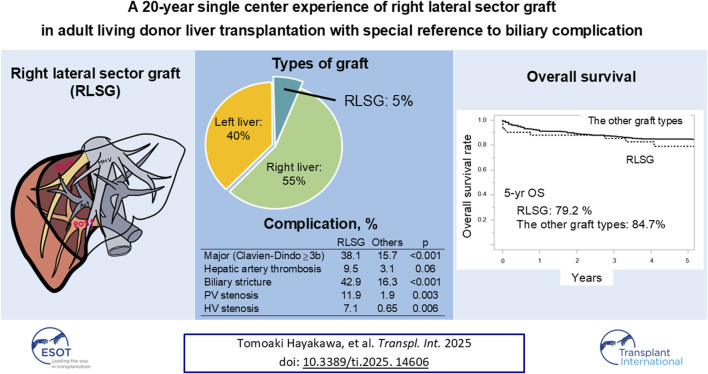

## Introduction

Living donor liver transplantation (LDLT) is often performed in countries with a scarcity of deceased donors [1–4]. The use of left liver grafts in adult LDLT was first reported in the 1990s, considering donor safety [5]. Subsequently, the procurement of right liver grafts began because of the graft volume advantage it comes with [3, 6]. Due to their volume advantage, right liver grafts have become a standard choice for transplantation [7]. However, since the right liver accounts for 60%–75% of the total liver volume, a relatively high incidence of mortality and postoperative liver failure has been reported among right liver graft donors [8], making this option inappropriate for donors whose right liver accounts for more than 70% of the organ’s total volume [9].

To address this limitation, our group has previously reported using right lateral sector grafts (RLSGs) for cases where a hemiliver transplant (i.e., right liver grafts or left liver grafts) is inappropriate [10]. Following our initial report, some institutions began to procure RLSGs for LDLT. While we have previously demonstrated the feasibility of RLSGs [11], there is still inadequate data on the long-term outcomes and the extent of donor candidate expansion because of the relatively rare indication and the technical difficulty associated with RLSG procurement. In this study, we aimed to report the feasibility and recipients’ long-term outcomes using RLSGs.

## Patients and Methods

### Data Source

In this study, we included 661 LDLTs performed from 2000 to 2021 at the University of Tokyo Hospital. During this study period, the selected graft types were 363 right liver grafts, 243 left liver grafts, 13 left lateral section grafts, and 42 RLSGs. The demographic and clinical data of patients were recorded prospectively and analyzed retrospectively. The detailed eligibility criteria for donors have been described previously [9]. These criteria were [1] age 20–65 years [2], donor-recipient relationship within 3 degrees of consanguinity or spousal, and [3] the absence of significant comorbidities. The study was conducted per the principles outlined in the Declaration of Helsinki and the Declaration of Istanbul. Demographic and clinicopathological characteristics were entered into a computerized database and retrospectively reviewed with the approval of the Graduate School of Medicine and Faculty of Medicine, The University of Tokyo Research Ethics Committee/Institutional Review Board (Approval number: 2140). Patients’ informed consent was obtained via opt-out on the website (http://plaza.umin.ac.jp/htokyotransplant/results/index.html).

### Graft Type Selection Criteria

**FIGURE 1:**
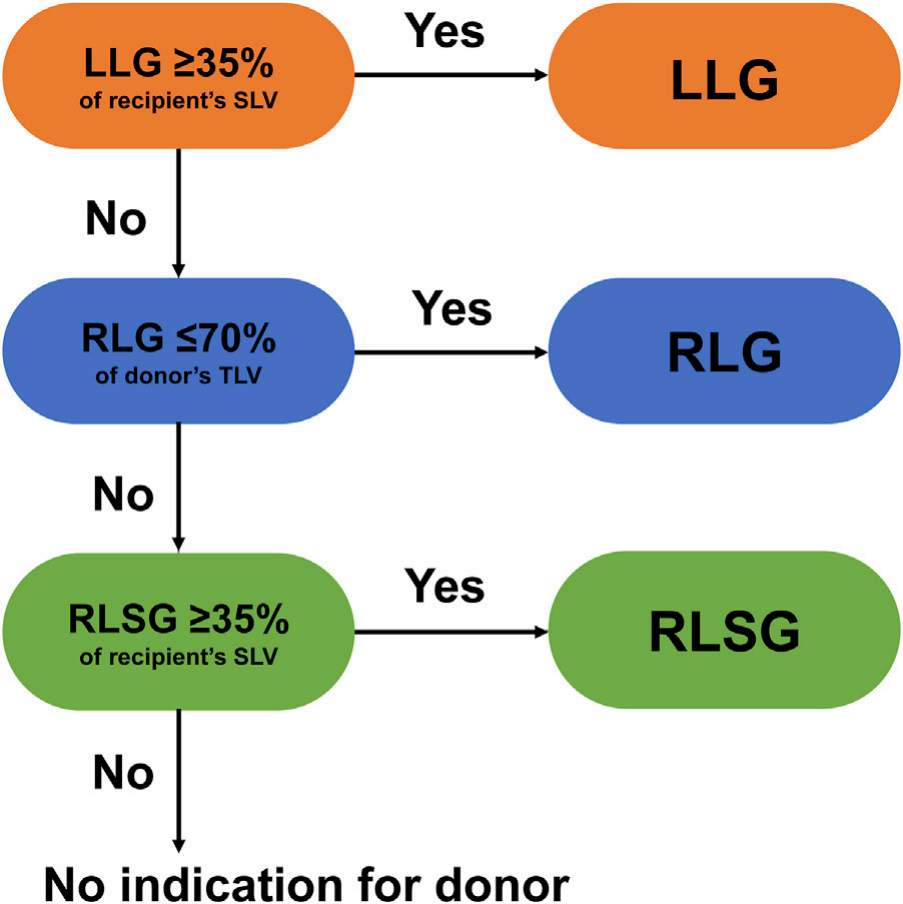
Algorithm for liver graft type selection at the University of Tokyo Hospital [9].

All donor candidates underwent abdominal ultrasonography to exclude fatty liver. A needle biopsy was performed if fatty liver was suspected, and regarding the degree of macrovascular steatosis, 10% was the criterion for donors. All donors had normal indocyanine green retention rates at 15 min. Graft types were selected based on the recipient’s standard liver volume (SLV) and the donor’s remnant liver volume. The graft selection algorithm is shown in Figure 1 [9]. First, left liver procurement was considered if the graft volume exceeded 35% of the recipient’s SLV. Second, right liver procurement was indicated if the donor’s remnant liver volume exceeded 30% of the total liver volume. Finally, we considered using an RLSG, provided the RLSG volume was more than 35% of the recipient’s SLV. Apart from the selection criteria, there were cases where RLSG was chosen due to anatomical factors, such as the unique branching of the portal vein (PV) in the right posterior section, even if the remnant liver volume met our eligibility criteria for right liver graft. While all donors underwent magnetic resonance cholangiopancreatography, when considering an RLSG, additional drip infusion cholecystocholangiography computed tomography was performed to rule out anomalies in the posterior bile duct that could make for unsuccessful transplantations. In nine out of the 42 RLSG cases, left liver graft or right liver graft could be selected; however, they used RLSG for favorable anatomical reasons.

### Donor Hepatectomy

The surgical procedure for living donor liver procurement has previously been described in detail [7]. A J-shaped incision was made to procure RLSGs. After cholecystectomy, we performed extrahepatic hilar dissection to isolate the right posterior hepatic artery and the PV (Supplementary Figure S1). The root of the right hepatic vein is exposed, and the short hepatic veins are ligated and divided. The middle and inferior right hepatic veins were preserved and divided before graft procurement. Next, the right posterior PV and artery were isolated and taped. The bile duct’s anatomy was confirmed using intraoperative cholangiography (Supplementary Figure S2). A demarcation line was identified after clamping the right posterior hepatic artery and PV. Finally, under Pringle’s maneuvers, liver resection was performed under ultrasound guidance.

### Recipient Surgery

The surgical procedure for recipients has previously been described in detail [10, 12, 13]. After total hepatectomy, the graft’s hepatic vein (HV) with patch plasty was anastomosed to the recipient’s inferior vena cava. When the graft contained a thick inferior right hepatic vein or middle right hepatic vein, we used the double inferior vena cava method to reconstruct these veins [14]. Subsequently, the right posterior PV was anastomosed to the recipient’s right or main PV with 6-0 polypropylene continuous sutures, and the plastic surgeons anastomosed the hepatic artery under a microscope with 7-0 nylon interrupted sutures. Then, duct-to-duct or hepaticojejunostomy anastomosis was performed for the bile duct [15, 16]. For the duct-to-duct anastomosis, the posterior wall was anastomosed with continuous sutures using 6-0 polydioxanone, while the anterior wall was anastomosed with interrupted sutures using 5-0 polydioxanone. Hepaticojejunostomy was performed using interrupted sutures with 5-0 polydioxanone for both walls. Stents were placed inside all anastomoses.

### Definition of Postoperative Complications and Posttransplant Management

According to the Clavien–Dindo classification, postoperative abdominal complications were recorded and graded [17]. In this study, complications graded as ≤3a were minor, while those graded as ≥3b were major. Because minor complications were frequent among LDLT recipients, we concentrated on the major complication rate and the mortality rate. The diagnosis of biliary leakage was based on the definition of the International Study Group of Liver Surgery [18]. Anastomotic biliary stricture was suspected based on a laboratory test or US/computed tomography images and confirmed by direct cholangiography via endoscopic retrograde cholangiography. We first diagnosed and treated the anastomotic biliary stricture via endoscopic retrograde cholangiography, followed by percutaneous transhepatic biliary drainage if necessary. We performed Doppler US twice daily for 2 weeks postoperatively to confirm adequate hepatic circulation and to detect hepatic artery thrombosis (HAT), PV stenosis, and HV stenosis. Protocol multidetector computed tomography was performed 1, 3, and 12 months after LDLT, and additional imaging studies were conducted based on clinical and laboratory findings. Biliary stents were removed 2–3 months after LDLT.

### Era Classification for the Technical Assessment

To assess the learning curve and technical evolution of this challenging procedure over time, we divided our 20-year experience with RLSG transplantation (2000–2021) into two equal periods: Era 1 (2000–2011) and Era 2 (2012–2021). This division enabled us to evaluate improvements in surgical times, complication rates, and postoperative outcomes as our surgical team accumulated experience with the technique.

### Statistical Analysis

All statistical analyses were performed using JMP 18.0.1 (SAS Institute Inc., Cary, NC) software. Continuous variables were presented as median values with interquartile ranges. Categorical variables were presented as frequencies and percentages and analyzed using the chi-square or the likelihood ratio test, as appropriate. Continuous variables were analyzed with the Mann–Whitney U test. Survival curves were drawn using the Kaplan–Meier method and compared with the log-rank test. All statistical analyses were two-tailed, and p-values of <0.05 were considered statistically significant.

## Results

Among 661 LDLTs performed between 2000 and 2021, 42 (6.4%) utilized RLSGs, with RLSG being the only viable option for 33 recipients (5%) according to our institutional criteria. The median follow-up period was 88 months (range: 0–272).

### Donor and Recipient Characteristics

RLSG donors (n = 42) had a median age of 42 years, with operation times of 525 min and blood loss of 543 mL (Table 1). The major complication (Clavien-Dindo grade ≥3b) rate was 4.8% (n = 2), with no mortality. Minor complications (Clavien-Dindo grade ≤3a) occurred in 33.3% (n = 14), with bile leakage being the most common (14.3%, n = 6). All donors recovered completely with no long-term sequelae. Recipients had a median age of 47 years and MELD score of 15.6. The median graft volume was 458g, representing 40.9% of the recipient’s standard liver volume (Table 1).

**TABLE 1:** Baseline characteristics and perioperative outcomes of right lateral sector graft donors and recipients.

Characteristics of donor	RLSG donor (n = 42)	Characteristics of recipient	RLSG recipient (n = 42)
Characteristics		Characteristics	
Age, yr	42 (30–49)	Age, yr	47 (32–56)
Sex (male/female)	25/17 (60.0/40.0)	Sex (male/female)	17/25
BMI, kg/m^2^	23.3 (21.2–25.4)	Adult/Child	37/5
Operative data		BMI, kg/m^2^	21.8 (19.2–24.6)
Operation time, min	525 (449–567)	MELD score	15.6 (12.6–20.9)
Blood loss, mL	543 (378–763)	Operative data	
Graft volume, g	458 (402–516)	Operation time, min	857 (745–968)
Hospital stay, days	14 (11–18)	Blood loss, ml	4,133 (2848–7,378)
Complication		Graft volume, g	458 (402–516)
Minor^a^	14 (33.3)	Graft-to-recipient SLV ratio, %	40.9 (36.6–46.1)
bile leakage	6 (14.3)		
Major^a^	2 (4.8)		
re-operation for postoperative bleeding	1 (2.4)		
Heart failure	1 (2.4)		
Death within 90 days	0 (0)		
Postoperative bile leakage	10 (23.8)		

Values are median (inter quartile range) or n (%).

^a^ minor complication was defined as Clavien-Dindo classification ≤3a, major complication was defined as ≥3b.

Abbreviations: AST, aspartate aminotransferase; ALT, alanine aminotransferase; PT-INR, prothrombin time-international normalized ratio; BMI, body mass index; MELD, model for end-stage liver disease; BMI, body mass index; MELD, model for end-stage liver disease.

### Comparative Outcomes

Compared to other graft types, RLSG recipients experienced significantly higher rates of major complications (38.1% vs. 15.7%, OR = 3.31, p < 0.001) and vascular complications (Table 2). These included portal vein stenosis (11.9% vs. 1.9%, OR = 6.84, p = 0.003), hepatic vein stenosis (7.1% vs. 0.65%, OR = 11.8, p = 0.006), and a trend toward higher hepatic artery thrombosis (9.5% vs. 3.1%, OR = 3.32, p = 0.06).

**TABLE 2:** Comparison of recipient characteristics and outcomes between right lateral sector and other grafts.

Characteristics	RLSG (n = 42)	Other graft types (n = 619)	OR	95% CI	p
Characteristics and graft data					
Age, yr	47 (32–56)	52 (43–58)	-	-	0.03
Sex, male/female (%)	17/25 (40.5/59.5)	336/283 (54.3/45.7)	-	-	0.08
BMI, kg/m^2^	21.8 (19.6–23.9)	22.7 (20.2–25.2)	-	-	0.12
MELD score	15.6 (12.7–20.5)	16.6 (13.0–21.0)	-	-	0.60
Operation time, min	857 (745–968)	833 (744–925)	-	-	0.69
Blood loss, ml	4,133 (2848–7,378)	4,830 (3,000–7,220)	-	-	0.45
Graft volume, g	458 (402–516)	518 (439–614)	-	-	<0.001
Graft volume/SLV ratio, %	41.1 (37.0–46.5)	44.0 (39.0–51.8)	-	-	0.02
GRWR, %	0.81 (0.69–0.93)	0.86 (0.73–1.02)	-	-	0.10
Postoperative complication					
Major^a^	16 (38.1)	97 (15.7)	3.31	1.71–6.40	<0.001
HAT	4 (9.5)	19 (3.1)	3.32	0.93–9.38	0.06
Biliary leakage	10 (23.8)	71 (11.5)	2.41	1.09–4.98	0.03
Biliary stricture	18 (42.9)	101 (16.3)	3.85	1.99–7.32	<0.001
PV stenosis	5 (11.9)	12 (1.9)	6.84	2.29–20.4	0.003
HV stenosis	3 (7.1)	4 (0.65)	11.8	2.27–55.5	0.006
90 days survival rate, %	90.5	96.6	3.00	0.84–8.37	0.08
Re-transplantation	1 (2.4)	4 (0.6)	3.89	0.20–27.0	0.30
Survival rates					
1-year survival rate, %	88.1	91.0	-	-	0.23
5-year survival rate, %	79.2	84.7	-	-	0.17

Values are median (interquartile range) or n (%).

^a^ Major complication was defined as Clavien-Dindo classification ≥3b.

Abbreviations: RLSG, right lateral sector graft; GRWR, graft-to-recipient body weight ratio; MELD, model for end-stage liver disease; HAT, hepatic artery thrombosis; HV, hepatic vein; PV, portal vein.

### Vascular Complications in RLSG

HAT occurred in 4 patients (9.5%), all in Era 1, with two cases resulting in early mortality. PV stenosis was observed in 5 patients (11.9%), occurring between postoperative day 3 and 1,618, with two cases resulting in mortality. Three cases (7.1%) of HV stenosis were reported, all in Era 2, occurring between postoperative day 119 and 776, with one case requiring stent placement (Table 3).

**TABLE 3:** Detailed summary of vascular and biliary stricture after right lateral sector grafts: onset, treatment, and outcomes.

Complication	Patient no.	Era	Postoperative day	Detail of treatment	Death within 90 days
Vascular complication					
HAT	1	1	3	Observation	Yes
	2		0	Re-operation	No
	3		19		No
	4		5		No
PV stenosis	1	1	1008	PTA	No
	2 (same as patient No.1 of HAT)		3	Observation	Yes
	3		1618	PTA	No
	4	2	6	Stent placement	Yes
	5		277		No
HV stenosis	1	2	776	Stent placement	No
	2		292		No
	3		119		No
Biliary stricture					
duct-to-duct	1	1	1535	External biliary drainage	No
	2		3	Re-operation	No
	3		816	Re-operation	No
	4		625	Inside stent placement	No
	5		216	Inside stent placement	No
	6		48	External biliary drainage	No
	7	2	107	External biliary drainage	No
	8		108	Inside stent placement	No
	9		84	Inside stent placement	No
	10		118	External biliary drainage	No
	11		72	Inside stent placement	No
	12		26	Inside stent placement	No
	13		95	Re-operation	No
	14		179	Inside stent placement	No
Hepaticojejunostomy	1	1	566	External biliary drainage	No
	2		790	Re-operation	No
	3		178	Re-operation	No
	4	2	139	Inside stent placement	No

Abbreviations: HAT, hepatic artery thrombosis; PV, portal vein; HV, hepatic vein; PTA, percutaneous transluminal angioplasty.

### Biliary Complications

Biliary complications were notably higher in RLSG recipients, with stricture rates of 42.9% versus 16.3% in other grafts (OR = 3.85, p < 0.001) and leakage rates of 23.8% versus 11.5% (OR = 2.41, p = 0.03). Univariate analysis identified graft bile duct length >4 mm as a significant risk factor for anastomotic biliary stricture (OR = 8.85, p = 0.03; Table 4).

**TABLE 4:** Univariate analysis of risk factors for biliary stricture in right lateral sector graft recipients.

Factor	No. of patients	No. of events	Univariate analysis		
			OR	95% CI	p
Patient factors					
Recipient age, yr	-	-	1.02	0.98–1.07	0.29
Sex	-	-			
Male	17	8	1.33	0.38–4.62	0.65
Female	25	10	reference		
BMI, kg/m^2^	-	-	1.06	0.90–1.25	0.50
MELD score	-	-	0.95	0.87–1.04	0.26
Operation time, min	-	-	1.00	0.99–1.00	0.17
Blood loss, ml	-	-	1.00	1.00–1.00	0.09
Graft volume, g	-	-	1.00	0.99–1.01	0.77
Graft volume/SLV ratio, %	-	-	0.97	0.91–1.05	0.47
GRWR, %	-	-	0.30	0.02–3.97	0.32
CIT	-	-	0.99	0.97–1.00	0.16
WIT	-	-	0.98	0.95–1.00	0.12
incompatible ABO	2	2	-	-	-
ACR	17	9	2.00	0.57–7.01	0.276
Biliary anastomosis					
Graft bile duct length, mm					
>4	6	5	8.85	1.25–179	0.03
≤4	36	13	reference		
Graft bile duct diameter, mm	-	-	1.23	0.78–1.95	0.31
Number of anastomoses	-	-	0.63	0.08–3.67	0.61
Duct-to-duct anastomosis	30	14	1.75	0.45–7.74	0.43
Hepaticojejunostomy	12	4	reference		

Abbreviations: OR, odds ratio; CI, confidence intervals; BMI, body mass index; MELD, model for end-stage liver disease; SLV, standard liver volume; GRWR, graft-to-recipient body weight ratio; CIT, cold ischemia time; WIT, warm ischemia time; ACR, acute cellular rejection; SLV, standard liver volume; GRWR, graft-to-recipient body weight ratio; CIT, cold ischemia time; WIT, warm ischemia time; ACR, acute cellular rejection.

Anatomical analysis revealed that low right lateral sectoral duct-common hepatic duct junction variants (n = 10) had the highest rates of biliary complications, with 60% developing strictures and 20% experiencing severe leakage. This variant was also associated with a higher proportion of grafts with bile duct length >4 mm (40%) compared to standard right hepatic duct formation (4.2%; Table 5).

**TABLE 5:** Comparison of biliary complications according to bile duct anatomical variants.

Anatomy	N	Biliary stricture, n (%)	Biliary leakage C-D ≥3a n (%)	Graft bile duct length >4 mm, n (%)
Standard RHD formation	24	9 (37.5)	2 (8.3)	1 (4.2)
RLSD–LHD junction	5	2 (40.0)	1 (20.0)	1 (20.0)
RLSD–LHD–RASD trifurcation	3	1 (33.3)	0 (0.0)	0 (0.0)
Low RLSD–CHD junction	10	6 (60.0)	2 (20.0)	4 (40.0)

Abbreviations: RHD, right hepatic duct; RLSD, right lateral sectoral duct; LHD, left hepatic duct; RASD, right anterior sectoral duct; CHD, common hepatic duct.

### Long-Term Outcomes

**FIGURE 2:**
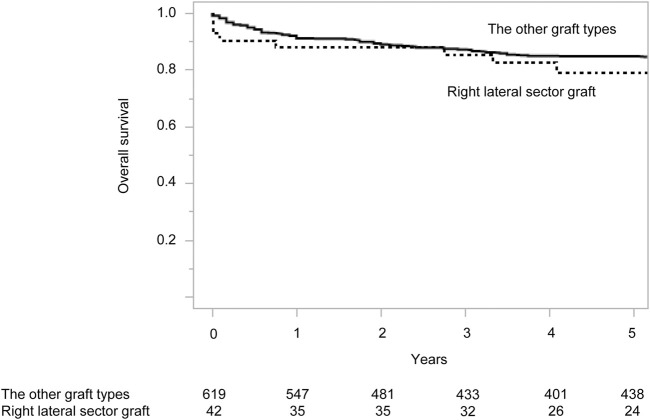
Overall survival of living donor liver transplantation recipients according to graft type. Numbers below the x-axis indicate the number of at-risk patients.

Despite higher complication rates, RLSG recipients showed comparable long-term outcomes to other graft types (Figure 2). The 90-day mortality rate was 9.5% (n = 4), with causes detailed in Supplementary Table S2. Re-transplantation was required in one RLSG recipient (2.4%) and in four recipients (0.6%) with other graft types (p = 0.30). The 5-year survival rate was 79.2% for RLSG recipients versus 84.7% for other graft types (p = 0.17).

### Era Analysis

Comparing Era 1 (2000–2011) to Era 2 (2012–2021), we observed significant improvements in operative times for both donors (563 vs. 440 min, p < 0.001) and recipients (933 vs. 724 min, p < 0.001). Notably, hepatic artery thrombosis occurred exclusively in Era 1 (15.4% vs. 0%, p = 0.04), while hepatic vein stenosis was only observed in Era 2 (0% vs. 18.8%, p = 0.01; Supplementary Table S1).

## Discussion

This study represents the largest single-center experience of RLSG in LDLT, comprising 42 cases over 20 years. Through this extensive experience, we identified that RLSG recipients had significantly higher rates of vascular and biliary complications compared with other graft types, despite successfully expanding the donor pool by 5%.

### Biliary Complications

Our analysis identified that graft bile ducts measuring >4 mm were significantly associated with anastomotic biliary stricture, particularly in cases with low right lateral sectoral duct-common hepatic duct junction. While surgeons traditionally consider this anatomical variant advantageous for RLSG procurement due to the ease of preserving a longer bile duct, our findings demonstrate that these longer ducts paradoxically increase the risk of stricture formation. This may be attributed to potentially compromised blood supply to the distal bile duct and/or increased risk of bile duct kinking at the anastomosis site. Based on these findings, we propose shortening the bile duct to maintain the anastomosis within the hilar plate, which may provide better blood supply to the anastomosis and potentially reduce the risk of bile duct kinking. However, we have not yet implemented this modified approach; therefore, its effectiveness remains to be evaluated in future studies.

### Vascular Complications

Our cohort demonstrated significantly higher rates of vascular complications, including HAT (9.5% vs. 3.1%), PV stenosis (14.3% vs. 1.9%), and HV stenosis (7.1% vs. 0.65%) compared to other graft types. The increased risk of HV stenosis might be attributable to unique anatomical features: the RHV runs beneath the graft’s raw surface, making it vulnerable to inflammatory changes, and the venous anastomosis alignment can be distorted during graft regeneration. Notably, while HAT occurred exclusively in Era 1, suggesting improvements in surgical technique over time, PV stenosis—not previously reported in RLSG studies—emerged as a significant complication requiring careful attention during reconstruction.

### Era Analysis and Technical Evolution

The comparison between Era 1 (2000–2011) and Era 2 (2012–2021) revealed significant improvements in surgical efficiency: operative times decreased substantially for both donors (563 vs. 440 min, p < 0.001) and recipients (933 vs. 724 min, p < 0.001). Additionally, donors’ hospital stays were significantly shorter in Era 2 (15 vs. 11 days, p = 0.006). Most notably, HAT, which occurred in 15.4% of cases in Era 1, was completely eliminated in Era 2 (p = 0.04), demonstrating the successful refinement of our surgical technique. However, the overall major complication rates remained comparable between eras (43.8% vs. 34.6%, p = 0.56), suggesting that while certain technical aspects improved over time, the intrinsic challenges of RLSG transplantation persist.

### Long-Term Outcomes and Donor Pool Expansion

Despite these technical challenges, our 1-year survival rate for RLSG recipients was 88.1%, consistent with previous reports (57.1%–100%) [19–21]. The 1-year survival rate for recipients of other graft types in our cohort was 91.0%, which did not differ significantly from RLSG recipients. Our 5-year survival rate of 79.2% for RLSG recipients was also comparable to previously reported RLSG survival rates (50%–100%) [20–22]. Among 661 LDLTs performed at our institution, RLSG was the only viable option in 33 cases, effectively expanding our donor pool by 5%.

This study has several limitations that are worth mentioning. Being a single-center retrospective study, its results still need to be validated in a multicenter prospective cohort study. The study period spans over 20 years, during which surgical techniques and perioperative management have evolved significantly, potentially affecting the outcomes and complication rates reported herein. These improvements include refinements in vascular reconstruction techniques, advancements in postoperative monitoring protocols, and developments in immunosuppressive regimens—all of which may have contributed to the differences observed between Era 1 and Era 2. In addition, there may have been selection bias in the decision-making process for RLSG, although we followed our institutional algorithm for graft type selection.

Thus, while RLSG transplantation successfully expanded our donor pool by 5%, our analyses revealed two critical findings that impact surgical strategy: first, preservation of longer bile ducts (>4 mm) significantly increases the risk of biliary complications, particularly in cases with low right lateral sectoral duct-common hepatic duct junction; second, RLSG recipients face a notably higher risk of portal vein stenosis than previously reported. These findings suggest that surgical success with RLSG could be improved by minimizing bile duct length and paying particular attention to portal vein reconstruction.

## Conclusion

RLSG transplantation represents a viable option for expanding the donor pool in living LDLT, demonstrating comparable long-term survival outcomes despite higher complication rates. While technical refinements over two decades have improved surgical efficiency and reduced certain complications, careful attention to anatomical factors - particularly biliary and vascular reconstruction - remains crucial for optimal outcomes. This procedure offers a valuable alternative for patients who would otherwise lack suitable donors, but its successful implementation requires surgical technique and careful patient selection.

## Data Availability

The raw data supporting the conclusions of this article will be made available by the authors, without undue reservation.

## Supplementary Material

SUPPLEMENTARY FIGURE S1 Intraoperative view of extrahepatic hilar dissection and isolation of the posterior branch of the right hepatic artery and right portal vein.

SUPPLEMENTARY FIGURE S2 Intraoperative cholangiography images showing the right hepatic duct’s anterior and posterior branches.

SUPPLEMENTARY FIGURE S3 Various patterns of bile duct branching: **(a)** Standard right hepatic duct formation, **(b)** right lateral sectoral duct-left hepatic duct junction, **(c)** right lateral sectoral duct-left hepatic duct-right anterior sectoral duct trifurcation, and **(d)** low right lateral sectoral duct-common hepatic duct junction.

## Abbreviations

HAT, hepatic artery thrombosis; HV, hepatic vein; LDLT, living donor liver transplantation; PV, portal vein; RLSG, right lateral sector graft; SLV, standard liver volume.
