# Supplementary material for: A 20-Year Single Center Experience of Right Lateral Sector Graft in Adult Living Donor Liver Transplantation With Special Reference to Biliary Complication
Source: Transpl Int. 2025 Jul 2;38:14606. doi: 10.3389/ti.2025.14606 (PMC12263453; doi:10.3389/ti.2025.14606)
Supplement: Supplementary file 3 [file Table1.docx]

| **Supplementary Table 1. Chronological comparison of surgical outcomes and complications in right lateral sector graft recipients: Era 1 (2000-2011) versus Era 2 (2012-2021)** | | | |
| --- | --- | --- | --- |
|  | Era 1, N = 26 (2000-2011) | Era 2, N = 16 (2012-2021) | p |
| **Donor** |  |  |  |
| Operation time, min | 563 (521-634) | 440 (376-524) | <0.001 |
| Blood loss, mL | 558 (420-763) | 395 (343-890) | 0.32 |
| Hospital stay, days | 15 (13.5-18.5) | 11 (8-15.8) | 0.006 |
| major complication | 1 (3.9) | 2 (12.5) | 0.56 |
| **Recipient** |  |  |  |
| Operation time, min | 933 (848-994) | 724 (638-794) | <0.001 |
| Blood loss, mL | 4180 (3026-7378) | 3812 (2603-7393) | 0.70 |
| Hospital stay, days | 47 (31-75) | 40 (29-64) | 0.57 |
| major complication | 9 (34.6) | 7 (43.8) | 0.56 |
| HAT | 4 (15.4) | 0 (0.00) | 0.04 |
| Biliary leakage | 4 (15.4) | 6 (37.5) | 0.11 |
| Biliary stricture | 9 (34.6) | 9 (56.3) | 0.17 |
| PV stenosis | 3 (11.5) | 2 (12.5) | 0.93 |
| HV stenosis | 0 (0) | 3 (18.8) | 0.01 |
| Abbreviations: HAT, hepatic artery thrombosis; PV, portal vein; HV, hepatic vein | |  |  |
