# Supplementary material for: A 20-Year Single Center Experience of Right Lateral Sector Graft in Adult Living Donor Liver Transplantation With Special Reference to Biliary Complication
Source: Transpl Int. 2025 Jul 2;38:14606. doi: 10.3389/ti.2025.14606 (PMC12263453; doi:10.3389/ti.2025.14606)
Supplement: Supplementary file 4 [file Table2.docx]

| **Supplementary Table 2. Cause of death and its correlation with vascular complications in right lateral sector graft recipients** | | | | |
| --- | --- | --- | --- | --- |
| No | Era | postoperative survival days | Detail | Correlation between anastomosed vessels and  primary cause of death |
| 1 | 1 | 6 | HAT, PV occlusion | Yes |
| 2 | 1 | 51 | ARDS | No |
| 3 | 2 | 15 | steroid-resistant ACR, PV stenosis | No |
| 4 | 2 | 12 | TMA, ARDS, fungal infection | No |
| Abbreviations: HAT, hepatic artery thrombosis; ARDS, acute respiratory distress syndrome; ACR, acute cellular rejection; PV, portal vein; TMA, thrombotic microangiopathy | | | | |
